# Grey matter volume reduction in the cerebellum of hip osteoarthritis patients: insights from pre- and postoperative VBM analysis

**DOI:** 10.1007/s11547-026-02184-2

**Published:** 2026-02-25

**Authors:** Francesca Caramia, Valentina Calistri, Maddalena Boccia, Alice Teghil, Costanza Giannì, Stefano Gumina, Marco Fiorelli, Alessandro Calistri

**Affiliations:** 1 Neuroradiology Unity, Department of Human Neurosciences, University of Rome Sapienza, Viale Dell’Università 30, 00185 Rome, Italy; 2 Vigne Nuove-Korian, Rome, Italy; 3 Department of Psychology, University of Rome Sapienza, Via Dei Marsi 78, 00185 Rome, Italy; 4 Cognitive and Motor Rehabilitation and Neuroimaging Unit, IRCCS Fondazione Santa Lucia, Rome, Italy; 5 Department of Anatomical, Histological, Forensic Medicine and Orthopaedic Science, Sapienza University of Rome, Piazzale A. Moro 3, 00185 Rome, Italy

**Keywords:** Osteoarthritis, Hip, Arthroplasty, replacement, Hip, Magnetic resonance imaging, Voxel-based morphometry, Gray matter, Neuroplasticity

## Abstract

**Purpose:**

This exploratory study aimed to investigate grey matter (GM) volume changes in patients with hip osteoarthritis undergoing hip joint arthroplasty using voxel-based morphometry (VBM), assessing pre- and postoperative differences and comparing findings with healthy controls (HC).

**Material and methods:**

Twenty-one patients with unilateral hip osteoarthritis before and after hip prosthesis insertion (PT group) and 16 HC were studied. All participants were right-handed and free from neurological or psychiatric conditions. Structural T1-weighted MRI scans were acquired at 3 T before and after surgery in the PT group, with an average interval of 112 days, and in the HC group. VBM analyses were conducted using the Computational Anatomy Toolbox (CAT12). Preoperative GM volume differences between PT and HC groups, as well as changes in PT before and after surgery, were analysed using statistical parametric mapping with family-wise error (FWE) correction.

**Results:**

Preoperative comparisons revealed a significant reduction in grey matter volume in the ipsilateral cerebellar Crus II in the PT group compared with HC (pFWE = 0.004). No significant GM volume differences were found between pre- and postoperative assessments in the PT group. Although all patients demonstrated marked clinical improvement at 1-month follow-up, MRI–clinical correlations could not be performed because clinical assessments and postoperative MRI were not acquired at the same time*.*

**Conclusion:**

The findings provide preliminary evidence of cerebellar GM changes in patients requiring hip prostheses, suggesting central nervous system involvement in chronic hip pathology. Interpretation is constrained by the modest sample size and by variability in postoperative imaging intervals. The absence of significant postoperative changes highlights the need for further research to explore the timeline and extent of neuroplastic recovery. These results underscore the importance of considering central adaptations in the management of peripheral joint disorders.

## Introduction

The implantation of hip prostheses is a widely adopted surgical intervention aimed at restoring mobility and alleviating pain in individuals with advanced hip joint pathology [1]. While the primary goal is mechanical correction, recent literature has emphasised the interaction between peripheral joint conditions and central nervous system mechanisms, particularly those involved in pain modulation and sensorimotor integration. These findings align with the growing recognition of the brain's role in musculoskeletal disease and recovery [2–4]. Recent scoping reviews have systematically summarised structural and functional brain alterations in knee OA, highlighting heterogeneous but convergent changes in pain-processing and sensorimotor networks [5–7].

Voxel-based morphometry (VBM), a neuroimaging method that allows voxel-wise comparisons of grey matter (GM) volume, provides a powerful tool for examining structural brain changes associated with peripheral joint conditions [8]. Previous studies have demonstrated alterations in brain morphology in chronic pain populations [6, 7, 9–12]; however, only limited data exist on the neuroanatomical impact of joint replacement surgery [13].

This study aimed to investigate GM volume changes in patients undergoing hip joint arthroplasty, both before and after surgery, compared to healthy controls (HC). We hypothesised that preoperative GM volume reductions might be present in regions involved in pain perception and sensorimotor control and that postoperative changes—if present**—**might reflect early neuroplastic recovery.

## Patients and methods

Twenty-one patients with clinically diagnosed hip osteoarthritis (HOA) and surgical indication for unilateral hip arthroplasty were recruited in the Orthopaedics Department at our institution. The sample included nine individuals with right-sided HOA (male:female, 8:1) and twelve with left-sided HOA (male:female, 11:1).

Inclusion criteria for patients were aged between 18 and 65 years; diagnosis of HOA according to the clinical classification criteria of the American College of Rheumatology [14]; and eligibility for surgical treatment with hip joint replacement. Exclusion criteria included secondary OA due to congenital or developmental diseases or inflammatory and autoimmune articular diseases, bilateral OA necessitating surgery within a year; other chronic pain conditions (e.g. knee osteoarthritis, chronic pelvic pain, fibromyalgia) and prior neurological or psychiatric disorders. Sixteen healthy controls (HC), matched by age, sex, and sociodemographic background, were recruited from the same geographical area. All MRI scans included in this work were originally acquired as part of routine preoperative clinical assessment for surgical planning. A small subset of examinations was performed after protocol submission but before formal approval; however, these scans were prescribed solely for clinical purposes and were not accessed or used for research until full Ethics Committee approval had been granted and written informed consent had been obtained for the research use of anonymised imaging data. Patients had been symptomatic for more than one year, many for multiple years before qualifying for total joint arthroplasty (mean body mass index 28.7 ± 2.3). None of the patients were on regular analgesic medication. Many had intermittently used nonsteroidal anti-inflammatory drugs (NSAIDs) and acetaminophen without sustained relief. Sixteen healthy participants (HC) were also enrolled (male:female, 13:3). All participants were right-handed. Participants of the hip prosthesis group (PT) and control participants (HC) did not differ significantly for age (PT: mean = 46.619 years, SD = 8.393; HC: mean = 45.00, SD = 9.695, t(35) = 0.544, *p* = 0.590, two-tailed).

Twenty patients underwent hip surgery with a resurfacing technique, and one with total hip arthroplasty. All surgeries were performed at the Orthopaedics Department by a single hip specialist.

### Clinical assessment

All patients underwent a clinical assessment before and after surgery including the Visual Analogue Scale (VAS), used to quantify pain intensity on a scale from 0 to 100, with higher scores indicating greater pain, the Harris Hip Score (HHS) to evaluate hip function, incorporating pain, mobility, and daily activity performance, ranging from 0 (worst) to 100 (best), the Oxford Hip Score (OHS) to assess hip-related quality of life using a 12-item questionnaire, with a total score ranging from 0 (worst) to 48 (best) [15–17] and UCLA activity score, which ranges from 1 (worst) to 10 (best)[18].

For statistical analysis, the Shapiro–Wilk test was used to assess the normality of data distribution. Normally distributed variables were summarised as mean ± standard deviation (SD), while non-normally distributed variables were reported as median (range). Preoperative and postoperative scores were compared using the paired t-test for normally distributed data and the Wilcoxon signed-rank test for non-normally distributed data. A significance threshold of p < 0.05 was considered statistically significant.

### Image acquisition and analyses

Anatomical T1-weighted MRI scans were obtained using a 3.0 Tesla Siemens Verio scanner (Siemens Medical Systems, Erlangen, Germany) with a high-resolution MPRAGE sequence. Imaging parameters were: voxel size 1 mm isotropic, repetition time (TR) of 1900 ms, echo time (TE) of 2.98 ms, flip angle of 9°, matrix size 256 × 256, 176 slices, and a field of view (FOV) of 256 mm.

Participants in the PT group underwent two MRI sessions, namely before and after surgery. To ensure the most reliable interpretation of any effects seen, our protocol included assessment and scanning of patients within one week prior to surgery (3 ± 2.8 days, mean ± SD), clinical assessment 1 month after surgery and postoperative MRI scanning at a median interval of three months (range: 49–254 days; median: 93 days; mean ± SD: 112 ± 60.3 days). The postoperative follow-up was chosen because pain and function have been consistently shown to improve significantly within the first 3 months [19, 20], and although improvements continue after this time period for a year and beyond [21] we intended to avoid natural grey matter atrophy seen in the ageing brain over this time period.

Voxel-based morphometric analysis was carried out using CAT12, implemented in SPM12. T1-weighted images were visually inspected for artefacts, then segmented into GM, white matter, and CSF. Normalisation to MNI space was achieved via high-dimensional Diffeomorphic Anatomical Registration Through Exponentiated Lie Algebra (DARTEL) normalisation, and the segmented GM images were checked for quality and smoothed using an 8-mm full-width at half-maximum (FWHM) kernel. All statistical analyses were performed on modulated, normalised grey matter maps, which reflect regional grey matter volume (GMV). Total intracranial volume (TIV) was included as a covariate. The statistical parametric maps resulting from all second-level analyses were thresholded at *p* < 0.05, corrected for multiple comparisons at the cluster level using family-wise error (FWE), after forming clusters of adjacent voxels surviving a threshold of *p* < 0.001 uncorrected. Given known limitations in posterior fossa registration in conventional VBM, we complemented the whole-brain voxel-wise analysis by re-examining the results within a cerebellar-inclusive mask derived from the Neuromorphometrics atlas. This procedure allowed us to verify that the significant cluster detected in the whole-brain analysis was entirely confined to cerebellar grey matter. Anatomical localisation of the cluster was further confirmed using the SPM SUIT atlas.

## Results

### Behavioural and clinical data

A significant improvement was observed across all assessed parameters at the postoperative follow-up. VAS scores decreased significantly, from a median of 70 (range: 40–90) preoperatively to 15 (range: 0–25) postoperatively (*p* < 0.001), reflecting substantial pain relief. HHS scores improved from a mean of 42.85 ± 12.61 preoperatively to 84.8 ± 4.18 postoperatively (*p* < 0.001), indicating enhanced hip function. OHS scores increased significantly, from a median of 18 (range: 12–32) preoperatively to 42 (range: 36–47) postoperatively (*p* < 0.001), suggesting a marked improvement in patient-reported quality of life and UCLA Activity Scores also increased significantly, from a median of 3 (range: 2–5) preoperatively to 4 (range: 3–7) postoperatively (*p* < 0.05), indicating a progressive return to physical activity. Collectively, these findings support the effectiveness of the intervention in reducing pain and enhancing hip function and quality of life within the first postoperative month.

### Voxel-based morphometry (VBM)

As a preliminary step, we compared the smoothed GM images of patients who underwent right- or left-sided hip surgery, before and after the intervention, performing two-independent sample t-tests. No significant difference before the intervention was found in GM volume between patients with hip osteoarthritis of the right or left hip. The comparison between groups after the intervention also did not highlight any significant difference between patients operated on the right or left hip. Since the GM volume of the two groups was comparable both before and after surgery, GM images of right-sided hip patients were left–right flipped, and patients were considered as a whole group in subsequent VBM analyses with brain side indicated as ipsi- or contralateral to the affected hip.

**Fig. 1:**
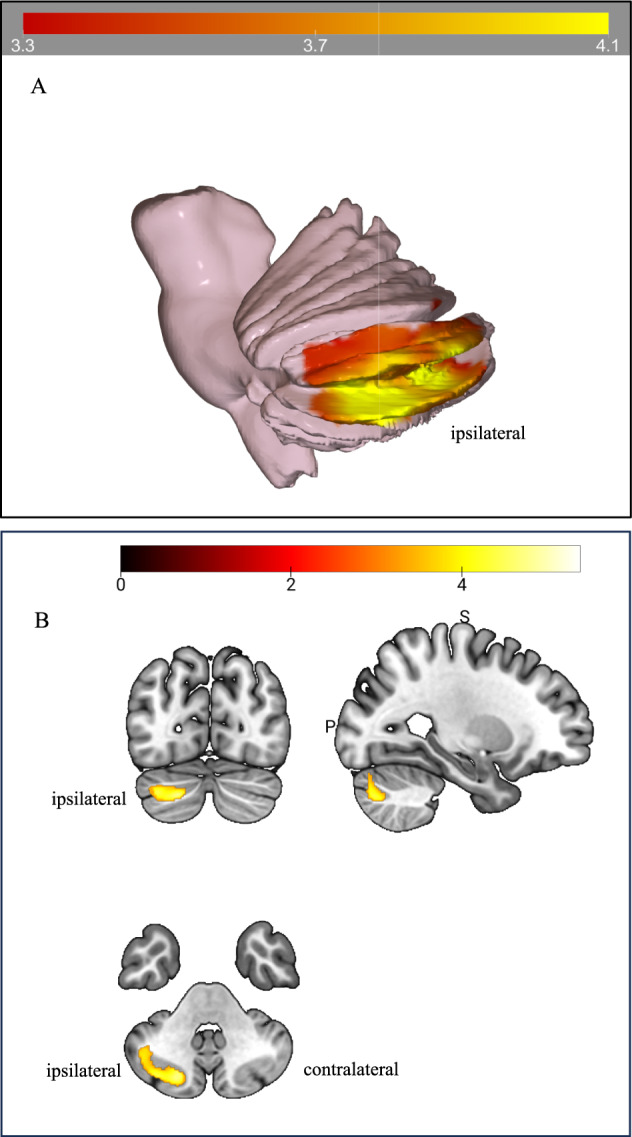
**A** Three-dimensional SUIT surface rendering of the cerebellum showing the cluster of significantly reduced grey matter (GM) volume in patients with hip osteoarthritis (PT) compared with healthy controls (HC). **B** Orthogonal slice views (axial, coronal and sagittal) of the same cluster. In all panels, the cluster is located in ipsilateral Crus II, corresponding to the symptomatic side after left–right flipping of right-hip patients. The peak statistical value is t = 5.48 (cluster-level pFWE = 0.004, peak-level *p* < 0.001 uncorrected). The red–yellow colour scale represents voxel-wise t-values from the VBM analysis thresholded at *p* < 0.001 uncorrected (t > 3.30) with a minimum cluster extent of 100 voxels. All visualisations were generated using the SUIT toolbox (SPM)

An independent-samples t-test was then performed to compare GM volume between HC and PT at the preoperative MRI assessment. A significant GMV reduction was found in the PT group compared to the HC group in a cluster located in the ipsilateral cerebellar Crus II (peak at MNI − 40, − 64, − 45) (t(1,34) = 5.48, pFWE = 0.004 at the cluster level; p uncorrected < 0.001 at the peak level), (Fig. 1A,B, Table 1). When inspecting the whole-brain results within a cerebellar-inclusive mask, the significant cluster in the ipsilateral cerebellum remained clearly identifiable and was entirely located in Crus II according to the SUIT atlas (peak *T* = 5.48, *Z* = 4.61, *p* < sub > FWE < /sub >  = 0.005; cluster size = 1237 voxels; peak coordinates − 40 − 64 − 45 mm, with additional peaks at − 22 − 78 − 39 mm and − 34 − 78 − 36 mm). No additional cerebellar clusters survived correction. Finally, a dependent-samples t-test was used to compare GM volume in the PT group before and after the intervention. No significant difference was found in GM volume between the two timepoints.

**Table 1:** Cluster statistics and MNI coordinates for the HC > PT contrast (gray matter volume reduction in patients). Statistical significance was set at cluster-level pFWE < 0.05, with voxel-level p < 0.001 uncorrected. Coordinates are reported in MNI space. The cluster was in the ipsilateral cerebellar Crus II

Cluster p(FWE-corr)	Cluster size (k)	T-value	Z-value	Peak p (uncorr)	MNI x	MNI y	MNI z
0.004	1259	5.48	4.61	< 0.001	– 40	– 64	– 45
		4.82	4.18	< 0.001	– 22	– 78	– 39
		4.58	4.01	< 0.001	– 34	– 78	– 36

To assess whether subtler effects might be present within the classical pain-processing network, we re-examined the whole-brain results within inclusive masks encompassing major pain-related regions (insula; anterior, mid- and posterior cingulate cortices; supplementary motor area; orbitofrontal, medial and lateral prefrontal cortices; primary and secondary somatosensory cortices; superior and inferior parietal lobules; supramarginal and angular gyri; and subcortical structures including nucleus accumbens, amygdala, putamen, thalamus, basal forebrain, and brainstem). No suprathreshold clusters emerged within these pain-related regions beyond the cerebellar finding described above.

## Discussion

The findings of this study provide preliminary evidence of altered grey matter (GM) volume in cerebellar regions in patients with hip osteoarthritis (HOA), particularly before surgical intervention. The observed reduction in GM within Crus II aligns with prior studies highlighting the cerebellum's involvement in both motor regulation and pain modulation. While structural differences in the cerebellum have been noted in various chronic pain populations, our results should be interpreted as exploratory, offering preliminary support for the involvement of posterior cerebellar regions in neuroplastic adaptations associated with long-standing peripheral joint pathology [11, 22–27].

The cerebellum plays a critical role in sensorimotor integration and motor behaviour, but its posterior regions, including Crus II, are also involved in higher-order cognitive processes and somato-motor functions, particularly when sensory input is compromised [28]. Recent research has expanded the understanding of the cerebellum’s diverse functions, extending beyond motor control to cognitive and affective processes. Functional topography studies have shown that cerebellar lobules VI-VII, particularly Crus I/II, are involved in higher-level cognitive tasks, such as language, spatial processing, and working memory [29]. Specifically, the posterior Crus II has been implicated in social mentalizing and emotional self-experiences [30]. Additionally, cerebellar lobules VI and VIIb exhibit multimodal processing for both motor control and pain, suggesting a role in pain-related adaptations of movement [31]. These interpretations are consistent with recent comprehensive reviews that have redefined the cerebellum as an active hub in pain processing, encompassing sensory–discriminative, cognitive, and affective dimensions of chronic pain [32, 33]*.*

The posterior cerebellum, particularly Crus I/II, has been associated with cognitive performance in working memory tasks, with load-dependent activity correlating with response times [34]. These findings support the segregation of cognitive and motor functions within the cerebellum and highlight its importance in various cognitive processes. Coombs and Misra investigated the integration of pain and motor processing in the human cerebellum, extending previous research that primarily focused on cortical areas such as the midcingulate cortex. Their study aimed to identify cerebellar regions activated during both pain and motor tasks and to determine whether this activation was confined to primary motor areas or extended to multimodal regions of the posterior cerebellum. They observed overlapping activity in lobules VI and VIIb, which persisted even when pain and motor tasks were combined or controlled. Functional connectivity analyses further revealed significant correlations between these cerebellar regions and key sensorimotor areas in the brain, including the anterior midcingulate cortex, supplementary motor area, and thalamus. These findings suggest that the posterior cerebellum, particularly lobules VI and VIIb, plays a crucial role in pain-related motor adaptations [31].

Interestingly, no significant GM volume differences were identified between pre- and postoperative MRI assessments in the PT group, suggesting that structural recovery may not occur within the early postoperative period. This lack of postoperative change should be interpreted cautiously, as it may reflect either an insufficient timeframe for neuroplastic recovery, limited statistical power, or persistent nociceptive and biomechanical factors following surgery [35]. The persistent GM reduction observed in our study aligns with previous findings that link Crus II to body representation and multisensory integration, particularly through its connections with the posterior parietal cortex, including the supramarginal gyrus, which is involved in non-action-related body representations [36]. The role of Crus II and adjacent cerebellar regions in sensorimotor adaptation and tool use learning has been previously demonstrated, suggesting that these areas contribute to the development of internal models for motor control and body-environment interaction [37–40]. A decrease in GM volume in cerebellar Crus II was observed in lower limb amputees who did not use a prosthetic limb, whereas no such decrease was found in those who used one [41]. While these studies have shown that cerebellar plasticity can be influenced by the extent of prosthesis use and phantom limb pain, our data do not allow us to conclude that hip surgery induces or fails to induce cerebellar recovery; rather, they suggest that such structural changes—if present—may require a longer postoperative interval to emerge [42].

Our study identified significant grey matter (GM) volume differences exclusively in the cerebellum, whereas previous studies have reported structural changes in other components of the salience/pain-processing matrix, such as the anterior cingulate cortex (ACC), insular cortex, and thalamus​ [6, 7, 9, 11, 13]. Several factors may contribute to these discrepancies, consistent with recent reviews highlighting the heterogeneity of structural and functional neuroplastic changes in osteoarthritis and with meta-analytic evidence showing highly variable and condition-dependent GM alterations across chronic pain syndromes [43]. First, our relatively small cohort consisted of patients undergoing either hip resurfacing or (one patient) total hip arthroplasty (THA). Hip resurfacing is typically performed in younger individuals with relatively preserved joint function, which may result in different patterns of central plasticity compared to those undergoing total THA [13]. Second, the use of a stringent whole-brain correction for multiple comparisons may have reduced sensitivity to subtle changes in smaller pain-processing regions previously identified through region-of-interest (ROI) analyses [10, 44]. Notably, in additional analyses where we re-examined the results within inclusive masks of classical pain-related regions (insula, cingulate cortices, somatosensory and parietal areas, and relevant subcortical nuclei), no suprathreshold clusters were detected, suggesting that any extra-cerebellar effects—if present—are likely small in magnitude. Third, methodological differences across studies—including acquisition parameters and preprocessing pipelines—may have affected the detection of small regional effects. Moreover, differences in analytic strategies—including whole-brain FWE-corrected approaches versus region-of-interest or small-volume correction methods—likely contribute to discrepancies between studies**.** Supporting this explanation is the absence of significant structural changes in osteoarthritis patients in a recent meta-analysis [45]. Lastly, it is possible that the cerebellum’s involvement in pain processing has been underappreciated, as emerging evidence suggests its role in integrating nociceptive input with motor adaptation and cognitive-affective processes [44].

Further studies with larger sample sizes, longer follow-up periods, and multimodal neuroimaging approaches are needed to clarify the distinct contributions of the cerebellum and pain matrix structures in the context of hip osteoarthritis and its surgical treatment. In particular, combining longitudinal structural and functional MRI would help to disentangle the temporal dynamics of clinical and neuroanatomical recovery.

Additionally, the absence of significant differences between right- and left-hip patients underscores the lateralized yet symmetrical involvement of cerebellar and other sensorimotor brain regions.

The limitations of this study include the relatively small sample size, the lack of long-term longitudinal data after surgery, and the temporal mismatch between postoperative clinical assessments and MRI examinations, which prevented meaningful MRI–clinical correlation analyses. Future studies should consider incorporating functional imaging techniques alongside VBM to explore the functional correlates of observed structural changes. Furthermore, investigating the relationship between GM volume changes and clinical outcomes, such as pain relief and functional recovery, would provide critical insights into the central effects of hip arthroplasty.

In conclusion, this study provides preliminary evidence of structural brain changes associated with hip joint pathology and its surgical treatment. These findings underscore the need for further exploration of the interplay between peripheral musculoskeletal disorders and central nervous system plasticity.
